# Content validation of the Myelofibrosis Symptom Assessment Form version 4.0 (MFSAF v4.0) in Janus kinase inhibitor–experienced patients with myelofibrosis

**DOI:** 10.1186/s41687-026-01047-8

**Published:** 2026-03-31

**Authors:** Katelyn Cutts, Anna Cardellino, Shiyuan Zhang, Kelesitse Phiri, Heather Gelhorn

**Affiliations:** 1 Evidera, Bethesda, MD, USA; 2 GSK, Collegeville, PA USA

## Abstract

Patients with myelofibrosis (MF) experience debilitating symptoms that negatively impact health-related quality of life and functioning. The Myelofibrosis Symptom Assessment Form version 4.0 (MFSAF v4.0) is a 7-item questionnaire assessing the burden of MF symptoms, including fatigue, night sweats, pruritus, abdominal discomfort, pain under the left ribs, early satiety, and bone pain. This study evaluated the content validity of the MFSAF v4.0 in Janus kinase (JAK) inhibitor–experienced participants with MF. Qualitative interviews were conducted with 20 symptomatic (MFSAF Total Symptom Score ≥ 10) participants from the US (*n* = 5) and UK, Germany, Italy, Poland, and Spain (*n* = 3 each). The interviews began with concept elicitation to allow patients to spontaneously report important symptoms. Next, content validation determined if the MFSAF v4.0 items and numeric rating scale (NRS) were relevant, important, and understandable. Overall, participants showed a clear understanding of the MFSAF v4.0, and no more than 3 (15%) mentioned that a given item was not relevant to their experience. Participants consistently felt that it was easy to select an answer on the NRS and often indicated that an improvement of 2 to 4 points was meaningful. These findings support the MFSAF v4.0 as a content-valid measure of selected disease-related symptoms for patients with JAK inhibitor–experienced MF.

## Introduction

Myelofibrosis (MF) is a myeloproliferative neoplasm characterized by clinical manifestations, including anemia, thrombocytopenia, splenomegaly, and debilitating symptoms [1]. The symptom burden experienced by patients is diverse, comprising constitutional (night sweats, fever, weight loss), spleen-related (abdominal pain, early satiety), and systemic (fatigue and pruritus) symptoms [2]. While some studies report that up to 30% of patients are asymptomatic at diagnosis, findings from internet-based surveys suggest that > 80% will have experienced ≥ 1 disease-related symptom prior to diagnosis [3, 4]. Currently, Janus kinase (JAK) inhibitors remain the standard of care [5], and clinical trials of the approved JAK inhibitors ruxolitinib [6, 7], fedratinib [8, 9], pacritinib [10, 11], and momelotinib [12–14] quantify symptom improvement as an established clinical trial endpoint. The MOMENTUM clinical trial of momelotinib, in particular, included symptom evaluation via the Myelofibrosis Symptom Assessment Form version 4.0 (MFSAF v4.0), a daily diary, 7-item patient-reported outcome (PRO) evaluating the severity of 7 MF-related symptoms over the past 24 hours [14].

The MFSAF v4.0 was developed by the Critical Path Institute’s PRO Consortium (MF Working Group), which reviewed past MF-related symptom questionnaires and developed the MFSAF v4.0 as a harmonized, consensus-based measure focused on the most relevant symptoms and measurement properties from previous questionnaires. This effort involved stakeholders from the US Food and Drug Administration (FDA), academia, and industry and included information from treatment-naive patients with MF to develop a new PRO based on the core MF symptoms of fatigue, night sweats, pruritus, abdominal discomfort, pain under the left ribs, early satiety, and bone pain [15]. Each item on the MFSAF v4.0 is self-reported daily on an 11-point numeric rating scale (NRS) from 0 (absent) to 10 (worst imaginable). The Total Symptom Score (TSS) ranges from 0 to 70, with higher scores indicating more severe symptoms. Although the MFSAF v4.0 has been evaluated in several studies both qualitatively and psychometrically, providing evidence of its construct validity and reliability [16, 17], the content validity of the tool has not been specifically assessed in those with prior JAK inhibitor experience. Given that treatment with JAK inhibitors may alter symptom burden, disease perception, and treatment, this study aimed to address that gap and build on prior studies by evaluating whether the MFSAF v4.0 adequately captures the distinct symptom profiles and perspectives of patients with MF and JAK inhibitor experience, ensuring its relevance and appropriateness for use in future research targeting this population.

## Methods

### Study design and interviews

Qualitative interviews were conducted remotely by trained interviewers with 20 self-reported JAK inhibitor–experienced (ruxolitinib, pacritinib, and fedratinib only, as momelotinib was investigational at the time of the survey) and symptomatic (MFSAF TSS ≥ 10) adults with a self-reported diagnosis of primary or secondary MF from the US (*n* = 5) and UK, Germany, Italy, Poland, and Spain (*n* = 3 each). Participants were recruited by email or social media posts, using an invitation with information about the study approved by an institutional review board (Salus IRB Study #23042–01). Eligible participants completed a remote concept elicitation and content validation interview for up to 60 minutes, which was audio recorded and transcribed for analysis. The interview guide was aligned with the FDA guidance for Selecting, Developing, or Modifying Fit-for-Purpose Clinical Outcome Assessments [18] and Methods to Identify What Is Important to Patients [19].

Concept elicitation allowed spontaneous reporting of symptoms associated with MF as well as frequency, severity, and changes in symptoms due to treatment. No specific symptoms were probed during this section. During the cognitive interview portion, each item of the MFSAF v4.0 was debriefed using probed questions to confirm comprehension (including describing the item in the participant’s own words), relevance, response to item, input on the response scale, and defining meaningful change (defined as how much improvement would need to be seen on the NRS). Participants were also asked about the ease and relevance of responding to each item using a 24-hour recall period. After debriefing on the MFSAF v4.0, participants were asked to confirm whether anything was missing that was important or relevant to their experience with MF.

### Data analysis

Final transcripts from the content validation interviews were entered into ATLAS.ti qualitative analysis software [20], which was designed for qualitative analysis of textual, graphical, audio, and video data. Translations of the MFSAF v4.0 for non-English language interviews were obtained by GSK via a third-party vendor and audio files were transcribed in the respective language and then translated to English. Analysis and saturation grids from the outputs were generated. In the saturation grid, concepts identified in each interview were analyzed in chronological order, and novel information was compared and tallied in each subsequent interview. Saturation was defined as the point at which no new themes, descriptions, concepts, or terms were introduced during any subsequent interview. For the analysis of sociodemographic and clinical questionnaire data, descriptive statistics were reported and summarized using R 4.0.5. Descriptive statistics were also used to summarize participants’ responses (from 0 to 10) to each item of the MFSAF v4.0 as well as their TSS (0 to 70, with higher scores corresponding to more severe symptoms). Continuous variables were summarized using means, medians, standard deviations (SD), and ranges; categorical variables were summarized by frequency statistics.

## Results

### Participant characteristics

Participants (*N* = 20) were, on average, 59.4 years of age (SD = 9.6), with an equal distribution of males and females. A majority (55%) were diagnosed with MF within the past year and almost all (95%) were on treatment at the time of the interview. On average, participants had 2.1 blood transfusions within 3 months prior to the interview, and 75% were transfusion independent, defined as ≤ 1 transfusion in any 1 month within the last 3 months (Table 1).

**Table 1:** Sociodemographic and self-reported key clinical characteristics of participants

Characteristics	Overall (*N* = 20)
**Age, years**	
Mean (SD)	59.40 (9.57)
**Sex, *n* (%)**	
Male	10 (50)
Female	10 (50)
**Country, *n* (%)**	
US	5 (25)
UK	3 (15)
Germany	3 (15)
Italy	3 (15)
Spain	3 (15)
Poland	3 (15)
**Employment status, *n* (%)**	
Full-time work	4 (20)
Part-time work	2 (10)
Homemaker	3 (15)
Unemployed	1 (5)
Retired	7 (35)
Disabled	2 (10)
Other	1 (5)
**Level of education, *n* (%)**	
College/university degree or higher ^**a**^	6 (30)
Other	14 (70)
**Health insurance, *n* (%)**^**b**^	
Employer provided	4 (20)
Self/private	3 (15)
Social	14 (70)
**Time since diagnosis, *n* (%)**	
≤ 12 months	11 (55)
12–60 months	4 (20)
> 60 months	5 (25)
**Type of MF, *n* (%)**	
Primary	12 (60)
Secondary	6 (30)
Don’t know	2 (10)
**Treatment experience, *n* (%)**	
Yes, currently	19 (95)
Yes, previously but not currently	1 (5)
**Time on current treatment, *n* (%)**	
≤ 6 months	11 (55)
6–12 months	3 (15)
12–60 months	3 (15)
≥ 60 months	2 (10)
Not applicable	1 (5)
**Diagnosis of anemia (since MF diagnosis), *n* (%)**	
Yes	18 (90)
No	0 (0)
Don’t know	2 (10)
**Diagnosis of anemia (current), *n* (%)**	
Yes	15 (75)
No	2 (10)
Don’t know	3 (15)
**Experience with blood transfusions due to MF, *n* (%)**	
Yes	16 (80)
No	4 (20)
**Transfusion status, *n* (%)**^**c**^	
TD	5 (25)
TI	15 (75)
**Number of blood transfusions over the last 3 months**	
Mean (SD)	2.12 (1.50)
Median (range)	2 (0–6)
**ECOG PS, *n* (%)**	
1	16 (80)
2	4 (20)
**Current treatments, *n* (%)**	
Ruxolitinib	16 (80)
Fedratinib	2 (10)
Pacritinib	1 (5)
Epoetin alfa	1 (5)
Hydroxyurea	1 (5)
Peginterferon alfa-2a	1 (5)
**Previous treatments, *n* (%)**	
Ruxolitinib	2 (10)
Fedratinib	1 (5)
Hydroxycarbamide	1 (5)
Thalidomide	1 (5)
Peginterferon alfa-2a	1 (5)
Danazol	1 (5)

^a^ Degree or higher includes any complete undergraduate or postgraduate degree

^b^ Non–mutually exclusive

^c^ TD was defined as ≥ 2 transfusions in any 1 month within the last 3 months; TI was defined as ≤ 1 transfusion in any 1 month within the last 3 months

ECOG, Eastern Cooperative Oncology Group; MF, myelofibrosis; PS, performance status; SD, standard deviation; TD, transfusion dependent; TI, transfusion independent

Participants had a mean MFSAF v4.0 TSS of 28.6 (SD = 16.6). Item 1 (fatigue) had the highest average score of 5.9 (SD = 2.4), with the remaining items having an average score of 2.3 to 3.7 (Table 2).

**Table 2:** MFSAF v4.0 descriptive statistics

Characteristics	Overall (*N* = 20)
**MFSAF TSS (range: 0–70)**^**a**^	
Mean (SD)	28.60 (16.57)
Min–max	10–54
Median (Q1–Q3)	21 (14–47)
**Item 1: Fatigue (range: 0–10)**^**a**^	
Mean (SD)	5.9 (2.36)
Min–max	1–10
Median (Q1–Q3)	6 (5–7)
**Item 2: Night sweats (range: 0–10)**^**a**^	
Mean (SD)	2.6 (2.58)
Min–max	0–8
Median (Q1–Q3)	2.5 (0–5)
**Item 3: Pruritus (range: 0–10)**^**a**^	
Mean (SD)	2.3 (2.41)
Min–max	0–8
Median (Q1–Q3)	2 (0–4)
**Item 4: Abdominal discomfort (range: 0–10)**^**a**^	
Mean (SD)	3.4 (3.08)
Min–max	0–9
Median (Q1–Q3)	3 (0–5)
**Item 5: Pain under left ribs (range: 0–10)**^**a**^	
Mean (SD)	2.8 (2.89)
Min–max	0–9
Median (Q1–Q3)	2.5 (0–4)
**Item 6: Feeling of fullness (range: 0–10)**^**a**^	
Mean (SD)	3.7 (3.07)
Min–max	0–9
Median (Q1–Q3)	4 (1–6)
**Item 7: Bone pain (range: 0–10)**^**a**^	
Mean (SD)	3.4 (2.52)
Min–max	0–7
Median (Q1–Q3)	3 (1–5)

^a^ Higher scores indicate more severe symptoms

max, maximum; MFSAF, Myelofibrosis Symptom Assessment Form; min, minimum; Q, quartile; SD, standard deviation; TSS, Total Symptom Score

### Concept elicitation

**Fig. 1:**
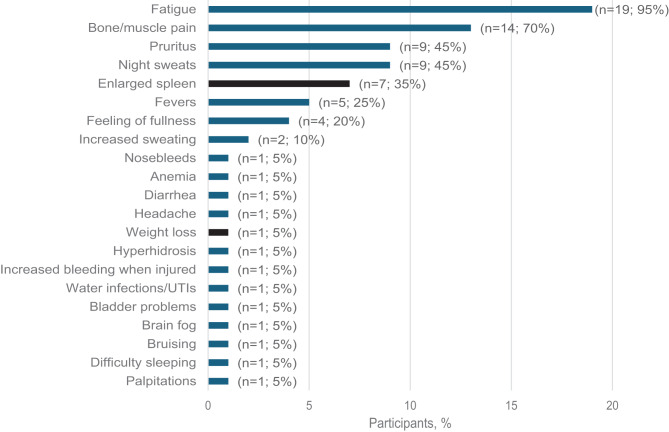
Concept elicitation: myelofibrosis-related signs and symptoms. Black corresponds to signs reported, and blue corresponds to symptoms reported by participants. UTI, urinary tract infection

During concept elicitation, the top spontaneously endorsed symptoms were fatigue (95%), bone/muscle pain (70%), night sweats (45%), and pruritus (45%). An additional 17 symptoms were spontaneously mentioned by participants, showing the heterogenous nature of the patient experience; however, many of these other symptoms were endorsed by only 1 or 2 patients each (≤ 10%). Saturation was achieved by the 15th interview for all spontaneously reported symptoms (Fig. 1).

### MFSAF v4.0 cognitive debriefing results

Overall, participants showed a clear understanding of the MFSAF v4.0, describing what each item meant to them in their own words and providing a rationale for their response. Participants described the day-to-day variability in their symptoms, and the majority reported that the 24-hour recall period was the most appropriate for capturing their experiences. While most of the items on the MFSAF v4.0 were understandable to all participants, 1 (5%) initially misinterpreted item 4 (“abdominal discomfort”), another (5%) suggested adding clarifying language to item 5 (“pain under left ribs”), and 2 (10%) asked for clarification on item 7 (“bone pain”). No more than 3 (15%) mentioned that a given item was not relevant to their individual experience (Table 3).

**Table 3:** MFSAF v4.0 cognitive debriefing results

Item	Understandable, *n/N* (%)	Relevant, *n/N* (%)	Easy to answer, *n/N* (%)	Notable quotes
Fatigue	20/20 (100)	19/20 (95)	13/17 (76)	*“It’s asking about when I experience fatigue that I can’t do anything. I can’t … sometimes it comes on so quickly that I can’t even walk upstairs or downstairs properly. My body just gives up and I have to rest.”*
Night sweats	20/20 (100)	17/20 (85)	13/13 (100)	*“This item is clearly asking about a noticeable symptom that comes along with this disease. It may seem otherwise, but it asks about the night sweats that forced us to wake up twice during the night to change the bed’s sheets. And it’s okay if this happens just once. But every night, on and on, it deteriorates our quality of life.”*
Pruritus	20/20 (100)	18/20 (90)	16/17 (94)	*“…it’s asking me in the last day if I had itching problems. I don’t really have itching problems.”*
Abdominal discomfort	19/20 (95)	19/20 (95)	16/16 (100)	*“It is clearly asking about the discomfort my spleen causes in my digestive system. It outlines it very clearly. Feeling pressure and bloating are the 2 main symptoms of a significant splenomegaly.”*
Pain under left ribs	20/20 (100)	17/20 (85)	12/15 (80)	*“Okay. In the past 24 hours how severe was the worst pain under your ribs on your left side? On a scale of 0 to 10. Absent … worst imaginable. I would place on a scale of between 8 and 9, I would go for 8. Because I can’t compare my pain to what other people feel, but I think put it on the scale of 8.”*
Early satiety	20/20 (100)	19/20 (95)	15/15 (100)	*“4 or 3 would actually be [great]. It would be [a] very great one [to move downwards], you know, I think when I actually eat a lot I get this kind of fullness within a few minutes. I don’t eat much, and I get full in a few minutes so I think, but I would find it easier to consume much more food.”*
Bone pain	18/20 (90)	17/20 (85)	11/13 (85)	*“Bone pain today would be 0 out of 10, but yes, I have had it in my journey, and the worst I probably have ever had it … worst day ever … probably, maybe I could equal a 7, my bones were … they felt broken.”*

MFSAF, Myelofibrosis Symptom Assessment Form

**Fig. 2:**
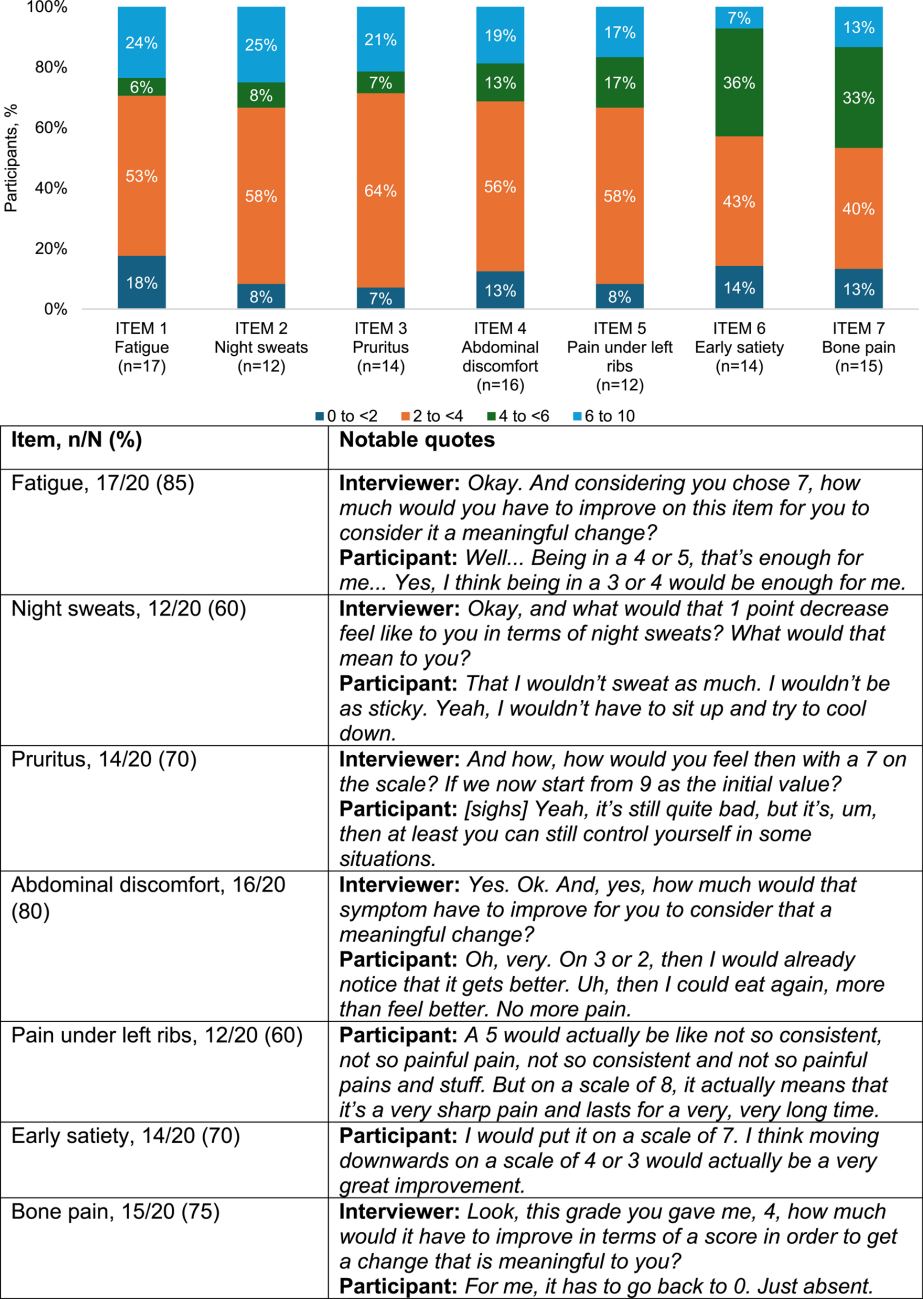
Meaningful change on the NRS. NRS, numeric rating scale

Participants consistently felt that it was easy to select an answer on the NRS using the 24-hour recall period and were able to differentiate between response options. When asked how they would define a meaningful change for each item based on their current response, participants most often indicated that an improvement of 2 to 4 points on the NRS for a symptom would be meaningful (Fig. 2).

At the conclusion of the interview, 15 participants were asked to give their overall feedback on the MFSAF v4.0; 5 participants were not asked due to time constraints. Almost half (*n* = 7/15; 47%) stated that no symptoms were missing, with 2 (13%) reporting symptoms missing, including dizziness and altered emotional state (*n* = 1/15; 7% each) and 6 (40%) mentioning missing signs and clinical information including anemia (*n* = 4/15; 27%), bad blood values (*n* = 2/15; 13%), hemoglobin levels (*n* = 2/15; 13%), colitis (*n* = 1/15; 7%), and vitamin deficiency (*n* = 1/15; 7%). Due to time constraints during the interview, 14 participants were also asked if they had any additional feedback on the MFSAF v4.0 before concluding the interview. One participant (7%) suggested an additional example for describing fatigue in item 1. Five participants (36%) had challenges with or suggestions about the recall period, including that it was complicated due to the limited 24-hour time frame and high variability in their symptom frequency (*n* = 2/14; 14%) and that the recall period be changed to 1 week (*n* = 2/14; 14%) or 1 month (*n* = 1/14; 7%). Overall, a majority stated that the recall period was easy to use for selecting a response when discussing each individual item.

## Discussion

This study builds on prior work by the PRO Consortium whereby existing MF PROs were reviewed and the MFSAF v4.0 was developed based on information from the FDA, academia, industry, and patients with MF who were treatment naive [15]. The current study strengthens the evidence that the MFSAF v4.0 is an appropriate measure of symptoms for patients with MF by demonstrating content validity in patients treated with a JAK inhibitor. Concept elicitation showed that most of the top spontaneously endorsed symptoms were also items in the MFSAF v4.0, further supporting the validity of the measure. Despite variability in symptom endorsement during concept elicitation as expected given the heterogeneity of patients’ experiences and lack of probes, most participants confirmed that the MFSAF v4.0 was still able to capture their experience with MF and no symptoms were missing. Participants consistently felt that it was easy to select an answer, were able to differentiate response options, and most often indicated an improvement of 2 to 4 points on the NRS would be meaningful.

Although items ask about symptom severity, participants often described meaningful change in terms of both a reduction in symptom severity and frequency. Participants described day-to-day variability in their symptoms, supporting the 24-hour recall period. Considering daily fluctuations in symptoms, using a weekly summary score to capture the average worst symptom experience within a week may also be beneficial. While a few participants reported some difficulty with selecting a response for an item or recommended changes, the results provide no evidence for necessary changes to the measure as feedback was below 15% of the sample.

Although larger sample sizes (i.e., additional interviews) can contribute to confidence in concept elicitation, there is a point at which additional sampling offers no new information, i.e., the point of saturation that was met by the 15th interview in this study [21, 22]. Additionally, the study may not be generalizable to all patients with MF due to participants being recruited through a third-party vendor. However, the study aimed to recruit a diverse sample across multiple countries and with varied transfusion requirements ranging from 0 to 6 transfusions within the past 3 months. A further potential limitation of the study was the requirement that participants have an MFSAF TSS ≥ 10 to be eligible, thus excluding patients with milder symptoms or those who may not have perceived their symptoms as relevant, and exposing patients to the MFSAF during screening, which may have biased subsequent cognitive debriefing. However, this criterion ensured that the participants included could meaningfully reflect and provide feedback on the concepts included in the MFSAF v4.0. As the MFSAF v4.0 is primarily intended for use in clinical trials focused on symptomatic patients and those eligible for intervention, validating the content of this tool in patients actively experiencing symptoms was of utmost importance.

This study confirms findings of the PRO Consortium [15], reinforces symptom assessment results of previous questionnaires [23], and is consistent with previous qualitative content validation studies in other MF patient populations [16]. Findings demonstrate that all items on the MFSAF v4.0 are comprehensible and relevant, with the NRS endorsed as an appropriate response scale. Although the MFSAF v4.0 is not designed to capture clinical information, it is notable that over one-quarter cited anemia as a clinical sign missing from the assessment, highlighting the need for alternative tools that measure this debilitating disease manifestation [24, 25]. Nevertheless, the results overall support that the content validity of the MFSAF v4.0 extends to patients with MF who have JAK inhibitor experience, showing that while patients with MF have heterogenous symptom experiences, their core symptoms align with the MFSAF v4.0 and the tool is a content valid measure that captures the most important concepts of interest.

## Data Availability

The datasets used and/or analyzed during the current study are available from the corresponding author on reasonable request. Information on GSK’s data-sharing commitments and requesting access to anonymized individual participant data and associated study documents can be found at https://www.gsk-studyregister.com/en/.

## Abbreviations

ECOG: Eastern Cooperative Oncology Group
FDA: US Food and Drug Administration
IRB: Institutional Review Board
JAK: Janus kinase
Max: Maximum
MF: Myelofibrosis
MFSAF v4.0: Myelofibrosis Symptom Assessment Form version 4.0
Min: Minimum
NRS: Numeric rating scale
PRO: Patient-reported outcome
PS: Performance status
Q: Quartile
SD: Standard deviation
TD: Transfusion dependent
TI: Transfusion independent
TSS: Total Symptom Score
UTI: Urinary tract infection
